# Primary Cutaneous Diffuse Large B-Cell Lymphoma, Leg Type: A Case Report

**DOI:** 10.7759/cureus.8651

**Published:** 2020-06-16

**Authors:** Ammar Al-Obaidi, Nathaniel A Parker, Khalil Choucair, Daniel Lalich, Phu Truong

**Affiliations:** 1 Internal Medicine, University of Kansas School of Medicine, Wichita, USA; 2 Pathology, Wesley Medical Center, Wichita, USA; 3 Hematology/Oncology, Cancer Center of Kansas, Wichita, USA

**Keywords:** primary cutaneous b-cell lymphoma, diffuse large b-cell lymphoma, leg type, myd88, lenalidomide, bruton’s tyrosine kinase, ibrutinib

## Abstract

Primary cutaneous diffuse large B-cell lymphoma, leg type, is an exceedingly rare and aggressive variant of primary cutaneous lymphoma. An 84-year-old male presented to an oncologist for new skin lesions on his abdomen and right thigh. Excisional biopsy followed by histopathology and immunohistochemistry confirmed the diagnosis of primary cutaneous diffuse large B-cell lymphoma, leg type. His clinical course was complicated by multiple relapses and refractory disease. Ultimately, he achieved complete response with fourth-line ibrutinib therapy. Due to the contentious nature of this disease, poor prognosis, and higher rates of recurrence, prompt identification and aggressive treatment are recommended. Given the different cellular pathways and genomic alterations identified in its carcinogenesis, various chemotherapy regimens and targeted immunotherapies have emerged as potential therapeutic options to halt disease progression and prevent future relapses.

## Introduction

Primary cutaneous lymphoma (PCL) is an extranodal lymphoma of the skin. The estimated annual incidence is 1 case per 100,00 persons in the Western countries. In contrast to nodal lymphoma, the majority of PCL cases are of T-cell origin, accounting for approximately 75% of all cutaneous lymphomas. Comprising approximately 25% of all PCL cases, primary cutaneous B-cell lymphoma (PCBCL) consists of three grossly similar, but clinicopathologic and molecularly distinct subtypes: primary cutaneous diffuse large B-cell lymphoma, leg type (PCDLBCL-LT), primary cutaneous marginal zone lymphoma, and primary cutaneous follicle center lymphoma [1,2]. Each subtype has a unique clinical presentation, pathology, prognosis, and treatment approach [3,4]. PCDLBCL-LT is rare, comprising only 4% of all cutaneous lymphomas. Most commonly affecting elderly women in the seventh decade of life, PCDLBCL-LT is an aggressive subtype that typically manifests as rapidly enlarging nodules on either a single leg or both. Even more unusual, PCDLBCL-LT can initially present at other dermatologic sites other than the lower extremities [4,5]. This report presents a unique and rare clinical case of relapsing and refractory PCDLBCL-LT in a male patient.

## Case presentation

**Figure 1:**
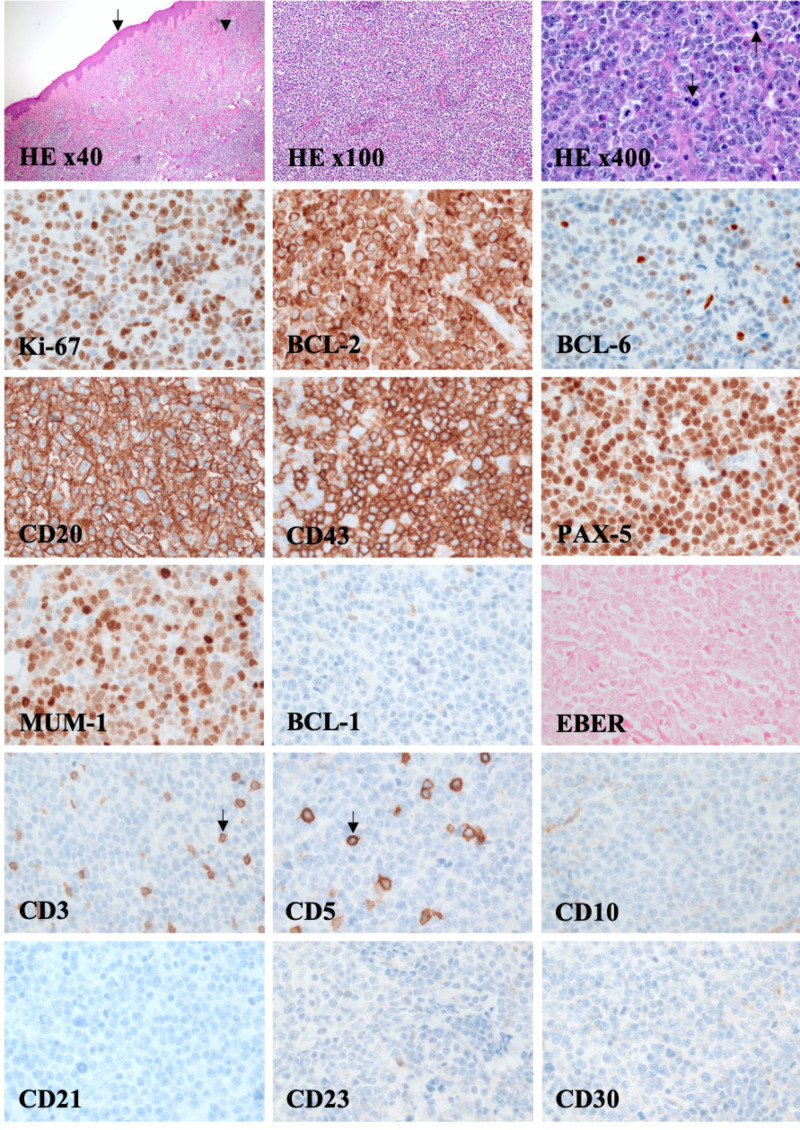
Excisional biopsy of the skin demonstrates primary cutaneous diffuse large B-cell lymphoma, leg type. Hematoxylin and eosin (HE) staining of epidermal (arrow) and dermal (arrowhead) skin layers. Microscopically, the cutaneous lymphoma is composed of monotonous, diffuse, non-epidermotropic infiltrate of confluent sheets of lymphoblasts extending throughout all of the dermis (HE x40 and x100). Mitotic figures are observed (arrow, HE x400). The cutaneous infiltrate of large lymphocytes lacks CD3 and CD5 immunoreactivity. Reactive CD3+/CD5+ T-cells (arrow) are relatively few. Neoplastic B-cells are strongly immunoreactive to cytoplasmic CD20, as well as nuclear PAX-5, BCL-2, and MUM-1 markers. Proliferation rate is high represented by Ki-67 staining. BCL-6 is partially positive by IHC, whereas CD10 and the follicular dendritic cell marker, CD21, is negative. Appropriately-controlled Epstein-Barr encoding region (EBER) studies by ISH show no nuclear staining or EBV-infected cells. This positive BCL-2, BCL-6, CD20, CD43, PAX-5, and MUM-1 and negative BCL-1, EBER, CD3, CD5, CD10, CD21, CD23, and CD30 IHC profile confirmed the diagnosis of primary cutaneous diffuse large B-cell lymphoma, leg type.

An 84-year-old male with a past medical history of chronic systolic heart failure with a reduced left ventricular ejection fraction of 40% presented to an outpatient oncology office with the chief complaint of small purple abdominal and right thigh skin lesions. Symptom onset began three months prior to his initial presentation. He underwent an extensive biochemical, cytologic, and radiologic workup which was primarily unremarkable. Excisional biopsy of the skin lesion was performed to provide the final pathological diagnosis. Immunohistochemical staining (IHC) was positive for CD20, CD43, PAX-5, MUM-1, BCL-2, and partial BCL-6 staining. Tissue specimens showed a lack of immunoreactivity with CD3, CD5, CD10, CD21, CD23, CD30, BCL-1, and EBER markers. A 75% proliferation index by Ki-67 staining was observed (Figure 1). MYC gene mutational testing by fluorescence in situ hybridization was negative. Ultimately, PCDLBCL-LT was confirmed.

He underwent the R-CVP chemotherapy regimen (rituximab, cyclophosphamide, vincristine, and prednisone) for six cycles followed by two weeks of hypofractionated radiotherapy (41.6 Gy in 3.2 Gy per fraction). Following treatment, surveillance imaging showed a complete response (CR). However, guideline-recommended serial monitoring ceased when the patient was lost to follow up.

The patient presented again four years later with three, two-week old violaceous cutaneous lesions on his left calf. PET/CT scan revealed multiple hypermetabolic cutaneous lesions in the distal lower extremities bilaterally without evidence of distant metastasis. Given his past medical history, excisional biopsy was performed due to concerns for recurrent disease. Microscopically, a diffuse and infiltrative population of large neoplastic cells harboring prominent nucleoli with vesicular chromatin was primarily localized to the deeper dermal layers and subcutaneous adipose tissue. Moreover, deeper adipose tissue, neurovascular bundle, and muscle fiber invasion was evident. Individual cell necrosis, as well as scattered mitotic figures, was observed. The IHC profile and cytogenetics were identical to the ones obtained in previous years. Thus, relapsing PCDLBCL-LT was diagnosed.

**Figure 2:**
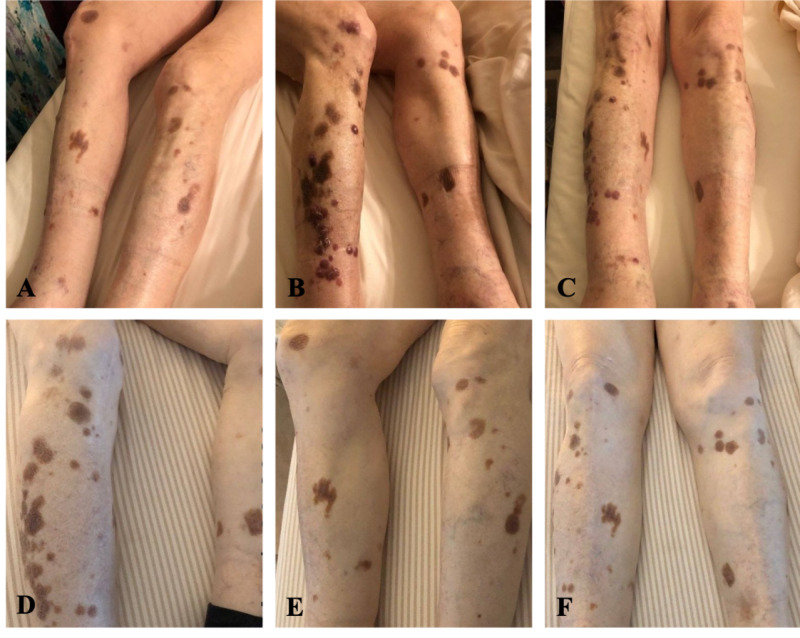
The clinical picture of primary cutaneous diffuse large B-cell lymphoma, leg type and its response to treatment. (A-C) Multiple violaceous, raised skin lesions involving the right lower extremity, before starting ibrutinib therapy. (D-F) Dissipating skin lesions approximately six months after initiating ibrutinib therapy.

The patient underwent combination chemotherapy of rituximab with bendamustine. Surveillance PET/CT scans after six months after initiating chemotherapy suggested refractory disease by showing multiple new hypermetabolic foci in the legs bilaterally and a thoracic lymph node. These findings were concerning for a second recurrence of cutaneous B-cell lymphoma. Third-line combination therapy of rituximab plus lenalidomide was started, but follow-up skin examinations and PET/CT scans demonstrated refractory and progressive disease (Figure 2A-2C). Due to the extent, refractory, and relapsing nature of his disease, a trial of Bruton's tyrosine kinase (BTK)-inhibitor therapy with ibrutinib was recommended. Routine surveillance by PET/CT scans and dermatologic examinations six months after starting fourth-line ibrutinib therapy have shown CR and skin lesion regression, respectively (Figure 2D-2F). Eight months after starting BTK-inhibitor therapy for this rare and aggressive cutaneous B-cell neoplasm, the patient was still in remission. However, he then expired due to natural, non-cancer related causes.

## Discussion

In contrast to nodal lymphomas, PCL cases are primarily of T-cell origin. PCBCLs represent about quarter of the cases of primary cutaneous lymphomas. Among the three PCBCL subtypes, the leg type variant is not only the most rare, but also characteristically has the higher rates of relapse and recurrence [4]. The median age at initial presentation is approximately 70 with a female predominance. As the name implies, PCDLBCL-LT preferentially involves the lower extremities with characteristic rapidly enlarging red to purple nodules. However, lesions are not necessarily confined to the lower extremities, as demonstrated in our case [4,5]. Regional lymph nodes and bone marrow represent the typical sites of extracutaneous spread. Furthermore, involvement of the central nervous system has also been reported [6-8].

An accurate diagnosis is essential in order to provide prompt treatment, especially considering the immunophenotypic overlap with other cutaneous lymphoma subtypes. Microscopically, PCDLBCL-LT is characteristically observed as diffuse, large monotonous B-cells in confluent sheets of immunoblasts with large round nuclei and prominent nucleoli in the deeper dermis and subcutaneous tissues with numerous mitotic figures. The epidermis may be spared, or surface ulceration may be present. Rarely, it can exhibit a mycosis fungoides-like pattern [9]. Tissue specimens predominantly show MUM-1 and BCL-2 expression [1]. Additionally, PCDLBCL-LT frequently expresses BCL-6, CD20, PAX-5, and CD79a. The germinal center marker CD10 is usually negative [7,8,10,11].

First-line treatment for PCLs is anthracycline-based chemotherapy combined with rituximab [10]. However, this is not always an option owing to age and comorbidity, as in our patient. Therefore, various rituximab plus polychemotherapy combinations are recommended, regardless of the clinical stage. Despite the lower incidence of PCDLBCL-LT, clinical trial data has been published that suggests the immunomodulatory agent, lenalidomide, is efficacious in management of relapsing and refractory PCDLBCL-LT [11]. Ibrutinib is a well-established therapeutic agent in a variety of B-cell lymphoproliferative disorders. Recently, ibrutinib has been shown to be one of the few agents to achieve complete response and durable remission rates in PCDLBCL-LT patients with relapsing and refractory disease [12].

## Conclusions

This report presented a unique and rare case of PCDLBCL-LT. The presence of violaceous nodules on the lower limbs should raise the suspicion of PCDLBCL-LT. However, lesions can involve areas of the body other than the lower limbs. Existing on the spectrum of extranodal cutaneous lymphomas, PCDLBCL-LT is an aggressive cutaneous lymphoma variant and its diagnosis, if delayed, can significantly affect prognosis. Thus, there is a need for increased clinical awareness. Clinicians should remain vigilant and have a low threshold for multi-specialty collaborations to enhance the likelihood of prompt identification and early, aggressive management. Although rituximab plus polychemotherapy is indicated for PCLs, no general consensus exists regarding optimal management for PCDLBCL-LT. However, complete responses and remission have been reported more than anecdotally following the administration of newer, immunomodulating and targeted therapies.
